# TAF1B depletion leads to apoptotic cell death by inducing nucleolar stress and activating p53-miR-101 circuit in hepatocellular carcinoma

**DOI:** 10.3389/fonc.2023.1203775

**Published:** 2023-08-14

**Authors:** Hang-fei Chen, Dan-dan Gao, Xin-qing Jiang, Hao Sheng, Qi Wu, Quan Zheng, Qiao-cheng Zhai, Lei Yuan, Ming Liu, Li-feng Xu, Mao-xiang Qian, Heng Xu, Jian Fang, Feng Zhang

**Affiliations:** ^1^ The 2nd Clinical Medical College, Zhejiang Chinese Medical University, Hangzhou, China; ^2^ Core Facility, The Quzhou Affiliated Hospital of Wenzhou Medical University, Quzhou People’s Hospital, Quzhou, China; ^3^ Department of Anus & Intestine Surgery, The First People’s Hospital of Jiande, Hangzhou, Zhejiang, China; ^4^ Department of Hepatobiliary Surgery, The Quzhou Affiliated Hospital of Wenzhou Medical University, Quzhou People’s Hospital, Quzhou, China; ^5^ The Joint Innovation Center for Engineering in Medicine, Quzhou People’s Hospital, Quzhou, China; ^6^ Institute of Pediatrics and Department of Hematology and Oncology, National Children’s Medical Center, Children’s Hospital of Fudan University, Institutes of Biomedical Sciences, Fudan University, Shanghai, China; ^7^ Center for Precision Medicine, The Quzhou Affiliated Hospital of Wenzhou Medical University, Quzhou People’s Hospital, Quzhou, China; ^8^ Department of Laboratory Medicine, West China Hospital, Sichuan University, Chengdu, Sichuan, China

**Keywords:** TAF1B, hepatocellular carcinoma, p53, nucleolar stress, RNA polymerase I, miR-101

## Abstract

**Background:**

TAF1B (TATA Box Binding Protein (TBP)-Associated Factor) is an RNA polymerase regulating rDNA activity, stress response, and cell cycle. However, the function of TAF1B in the progression of hepatocellular carcinoma (HCC) is unknown.

**Objective:**

In this study, we intended to characterize the crucial role and molecular mechanisms of TAF1B in modulating nucleolar stress in HCC.

**Methods:**

We analyzed the differential expression and prognostic value of TAF1B in hepatocellular carcinoma based on The Cancer Genome Atlas (TCGA) database, tumor and paraneoplastic tissue samples from clinical hepatocellular carcinoma patients, and typical hepatocellular carcinoma. We detected cell proliferation and apoptosis by lentiviral knockdown of TAF1B expression levels in HepG2 and SMMC-7721 cells using clone formation, apoptosis, and Western blotting (WB) detection of apoptosis marker proteins. Simultaneously, we investigated the influence of TAF1B knockdown on the function of the pre-initiation complex (PIC) by WB, and co-immunoprecipitation (Co-IP) and chromatin immunoprecipitation (ChIP) assays verified the interaction between the complexes and the effect on rDNA activity. Immunofluorescence assays measured the expression of marker proteins of nucleolus stress, fluorescence *in situ* hybridization (FISH) assays checked the rDNA activity, and qRT-PCR assays tested the pre-rRNA levels. Regarding molecular mechanisms, we investigated the role of p53 and miR-101 in modulating nucleolar stress and apoptosis. Finally, the impact of TAF1B knockdown on tumor growth, apoptosis, and p53 expression was observed in xenograft tumors.

**Result:**

We identified that TAF1B was highly expressed in hepatocellular carcinoma and associated with poor prognosis in HCC patients. TAF1B depletion modulated nucleolar stress and apoptosis in hepatocellular carcinoma cells through positive and negative feedback from p53-miR-101. RNA polymerase I transcription repression triggered post-transcriptional activation of miR-101 in a p53-dependent manner. In turn, miR-101 negatively feeds back through direct inhibition of the p53-mediated PARP pathway.

**Conclusion:**

These findings broaden our comprehension of the function of TAF1B-mediated nucleolar stress in hepatocellular carcinoma and may offer new biomarkers for exploring prospective therapeutic targets in HCC.

## Introduction

Hepatocellular carcinoma (HCC) remains a significant health challenge worldwide, and its incidence increases yearly (1, 2). In 2020, hepatocellular carcinoma was the third most common cause of cancer-related death and ranked sixth in incident cases (3). It is estimated that in 2030, more than 1 million patients will die from liver cancer annually (2). However, surgical resection and liver transplantation are the primary options for treating HCC. However, it is not indicated for patients with advanced HCC. In addition, tumor metastasis and recurrence are the main reasons for poor prognosis in HCC patients. Therefore, the treatment of HCC still needs novel therapeutic targets and regimens.

Ribosome biogenesis is a well-known feature of cell growth and proliferation (4). The nucleolus is a subnuclear compartment well recognized for its involvement in ribosome production. Ribosome biogenesis involves several processes, beginning with RNA polymerase I (Pol I) transcription and progressing through pre-rRNA processing and ribosomal assembly (5). Various cellular stressors that disrupt ribosome biogenesis will lead to nucleolar stress, and any error in the steps implicated in ribosome biosynthesis may lead to nucleolus stress, featuring modifications in nucleolus morphology and function (6, 7). In contrast, nucleolar volume increases in tumor cells, which stimulates the massive expansion of tumor cells by promoting ribosome biogenesis and reducing nucleolar stress (8). Some studies have indicated that nucleolus size may be available as a prognostic marker to determine tumor progression. In addition, nuclear ribosome biosynthesis has been demonstrated to facilitate cell cycle progression in breast cancer and glioblastoma (9). Suppression of ribosome biosynthesis activates the tumor suppressor protein p53 expression through the nucleolus stress response (10). These phenomena indicate that ribosome biosynthesis and nucleolar stress may be critical links in governing tumor progression.

We investigated the molecular function of TAF1B, a TBP-associated factor (TAF) that regulates ribosome biosynthesis. Our study identified a nucleolar stress disorder mediated by the Pol I transcriptional machinery and its transcriptional cofactors that facilitates hepatocellular carcinoma progression. Pol I has a low affinity for promoter sequence elements; proper recruitment at the promoter of the rDNA gene requires the assistance of specific transcription factors (11). The primary transcription co-factors of the pre-initiation complex (PIC) for Pol I in mammals are selectivity factor 1 (SL1), chromatin-bound upstream binding factor (UBF), and RRN3 (12, 13). In mammalian cells, Pol I transcription is dependent on SL1, which is a complex of TBP, TAF12, and at least four Pol I-specific TAFs: TAF1A (TAF_I_48), TAF1B (TAF_I_63), TAF1C (TAF_I_110), and TAF1D (TAF_I_41) (14). In addition, UBF binds throughout the rDNA in cells as a nucleolar scaffold protein and promotes the decondensation of rDNA chromatin (15). SL1 interacts cooperatively with UBF at the rDNA promoter through its TAF1A and TBP subunits. This stabilizes the association of UBF with the PIC and facilitates rDNA transcription (16). RRN3 interacts directly with Pol I and SL1 subunits TAF1B, TAF1C, and TAF1D. By tying Pol I to other essential promoter-binding factors and aiding polymerase recruitment to the PIC at the rDNA promoter, RRN3 is crucial in directing specific transcription initiation at rDNA genes (17). Genetic inactivation of PIC component RRN3 has been reported to lead to nucleolar disruption, cell cycle arrest, and apoptotic cell death (18).

Although SL-1 is required to initiate ribosomal RNA synthesis by RNA polymerase I, its function in cancers is not fully understood. In this study, we investigated the role of TAF1B, a key component of SL-1, in HCC. We observed that inhibition of TAF1B caused nucleolar stress and p53-mediated apoptosis. HCCs are susceptible to p53-mediated apoptosis, but depletion of TAF1B inhibits rRNA processing and ribosomal protein gene transcription. At a molecular level, we proved that impaired rRNA synthesis elicited a DNA damage response, and rDNA damage contributed to apoptosis in HCC. Our findings suggest that TAF1B might be a novel target for treating HCC.

## Materials and methods

### Patients and tissue samples

Tissue samples were gathered from patients with hepatocellular carcinoma who had undergone radical resection. Tumors and paired paracancerous tissues collected were used for Western blotting (WB) and immunohistochemical analysis. Each patient signed an informed consent form for sample collection.

### Cell culture

HEK293T, L-02, and HCC cell lines (HepG2, SMMC-7721, Huh7, SK-Hep-1, Bel-7404, MHCC-97H, and Hep3b) were purchased from American Type Culture Collection (Manassas, VA, USA). L-02, HepG2, Huh7, SK-Hep-1, and Hep3b cell lines were cultured in Dulbecco’s Modified Eagle Medium (DMEM; #8120448, Thermo Fisher Scientific, Shanghai, China). SMMC-7721 and Bel-7404 were cultured in Roswell Park Memorial Institute (RPMI) 1640 medium (1640, #11875500, Thermo Fisher Scientific, Shanghai, China). HEK293T was cultured in Minimum Essential Medium (MEM; #11095072, Thermo Fisher Scientific, Shanghai, China). All cells were maintained in a medium supplemented with 10% fetal bovine serum (FBS; #10099, Gibco, Shanghai, China) in a humidified chamber with 5% CO_2_ at 37°C.

### Lentivirus production and transduction

The method for lentivirus production and transduction was introduced previously. The oligos of shTAF1B RNAs (#1, #2, #3, #4, #5, and #6) were synthesized in Genewiz (Suzhou, China) and cloned in pLKO.1 lentiviral vector. The target sequences of shRNA are listed in **Table 1**. Specifically, shRNAs mixed along with the psPAX2 and pMD2.G plasmids were co-transfected into HEK293T cells with 80%–90% confluence in a six-well plate using Lipofectamine 3000 reagent (#L3000015, Thermo Fisher Scientific, Shanghai, China) to generate lentiviral particles for gene transduction. Twenty-four hours after transfection, virus-containing supernatants were collected and centrifuged at 300 g for 5 min to remove suspended HEK293T cells. The supernatants were mixed with polybrene at a final working concentration of 10 μg/mL to infect the target cells. At 48 h post-transduction, the target cells were selected with 10 μg/mL of puromycin for 2 days and then used for subsequent experiments. MiR-101 inhibitor (inhibit-miR-101), U6 primer, and miR-101 primer were purchased from GenePharma (Shanghai, China).

**Table 1:** The targeting sequences of shRNA.

Name	5’-3’ sequences
shTAF1B RNA#1	GCCACAATGTTACAGAGAGAT
shTAF1B RNA#2	GCAGGTGAGCTTCATTTGATT
shTAF1B RNA#3	CCTACGGTATTAGAAGATAAT
shTAF1B RNA#4	GCCTTAAAGAACCTTGGAGTA
shTAF1B RNA#5	CCTACGGTATTAGAAGATAAT
shTAF1B RNA#6	GCAGGTGAGCTTCATTTGATT

### Immunohistochemical staining and TUNEL

For immunohistochemical staining, paraffin-embedded sections (4 μm thick) were deparaffinized with xylene, hydrated with decreasing concentrations of ethanol, processed in 10 mmol/L of citrate buffer (pH 6.0), and heated in a microwave oven for 15 min for antigen retrieval. Tissue sections were treated with 3% hydrogen peroxidase in phosphate-buffered saline (PBS) for 10 min to block endogenous peroxidase activity. Sections were blocked with 5% goat serum for 30 min and incubated with primary antibodies overnight at 4°C. Primary antibodies used for immunohistochemistry included anti-TAFIB (1:150, #PA5-112957) acquired from Thermo Fisher Scientific (Shanghai, China) and anti-p53 (1:500, #2524) obtained from Cell Signaling Technology (Danvers, MA, USA). MaxVision™ HRP-Polymer IHC Kit (MXB Biotechnologies, Fuzhou, China) developed the signal. Hematoxylin counterstained the nucleus. According to the manufacturer’s instructions, TUNEL staining uses a Colorimetric TUNEL Apoptosis Assay Kit (C1091, Beyotime, Shanghai, China). First, paraffin-embedded sections (4 μm thick) were deparaffinized with xylene and hydrated with decreasing ethanol concentrations. Then, 20 μg/mL of DNase-free proteinase K was added and incubated for 15–30 min at 20°C–37°C, rinsed with PBS, and incubated with 3% H_2_O_2_ in PBS for 20 min at room temperature. Biotin labeling solution was prepared and incubated with sections at 37°C for 60 min in the dark. Cells were rinsed with PBS and incubated with streptavidin–horseradish peroxidase (HRP) working solution and DAB color-developing solution. Next, sections were dehydrated with increasing concentrations of ethanol to xylene. Finally, tissue sections were observed with the microscope (DMEX30, Sunny Optical Technology, Zhejiang, China).

### Colony formation assay

The shTAF1B lentivirus was transfected into HepG2 and SMMC-7721 cells, and the cells were inoculated in six-well plates (2,000/well) and incubated for 10 days. Cells were then immobilized in 4% paraformaldehyde for 15 min, washed three times with PBS, and stained with 1% crystal violet for 15 min. The colony counts were conducted using ImageJ software.

### Apoptosis assay

Apoptotic cells were detected using the FITC Annexin V Apoptosis Detection Kit with PI (#640914, BioLegend, San Diego, CA, USA). The cells were harvested, washed with cold PBS, and re-suspended with cold 1× binding buffer, according to the manufacturer’s instructions. In a microcentrifuge tube, 100 µL of cell suspension was transferred and added with 5 µL of FITC Annexin V. Then, 10 µL of propidium iodide solution was added. The cells were gently vortexed and incubated for 15 min at room temperature (25°C) in the dark. Next, 400 µL of Annexin V Binding Buffer was added to each tube. At least 10,000 cells per sample were collected using an LSRFortessa Cell Analyzer (BD Biosciences, San Jose, CA, USA) and analyzed with FlowJo software (Ashland, OR, USA).

### Western blotting

Cells were collected and lysed with 1× sodium dodecyl sulfate (SDS) buffer supplemented with phenylmethylsulfonyl fluoride (PMSF) and 1% phosphatase inhibitor (Beyotime), and then the protein content of different fractions was assayed using the bicinchoninic acid (BCA) method. The cell lysates were boiled for 10 min and centrifuged at 16,000 *g* for 10 min at 4°C to remove cellular debris. Equal amounts of proteins (25 μg) were separated on 10% SDS–polyacrylamide gel electrophoresis (SDS–PAGE) gels, transferred to polyvinylidene difluoride (PVDF) membranes (Millipore, Billerica, MA, USA), closed with 5% bovine serum albumin (BSA) for 1 h at room temperature, and incubated overnight at 4°C with primary antibodies. Primary antibodies, including anti-TAF1B (1:500, #PA5-112957) and anti-TAFID (1:1,000, #PA5-25509), were purchased from Thermo Fisher Scientific (Shanghai, China). Anti-Bcl-2 (1:1,000, #4223), anti-Bax (1:1,000, #5023), anti-Caspase-9 (1:1,000, #9502), anti-Cleaved-Caspase-9 (1:1,000, #7237), anti-Caspase-3 (1:1,000, #9662), anti-Cleaved-Caspase-3 (1:1,000, #9661), anti-Caspase-7 (1:1,000, #12827), anti-Cleaved-Caspase-7 (1:1,000, #8438), anti-p53 (1:1,000, #2524), and anti-PARP (1:1,000, #9542) were purchased from Cell Signaling Technology (Danvers, MA, USA). Anti-TAFI p48 (TAF1A) (1:500, #sc-393600) and anti-TAFI p110 (TAF1C) (1:500, #sc-374551) were purchased from Santa Cruz Biotechnology (Dallas, TX, USA). Anti-TAF12 (1:1500, #ab229487) and anti-TBP (1:800, #ab818) were purchased from Abcam (Cambridge, UK). The membranes were then blotted with HRP-conjugated secondary antibodies for 1 h at room temperature. Chemiluminescent signals were acquired using the Tanon 4200SF system (Tanon Biotechnology, Shanghai, China).

### Co-immunoprecipitation

For co-immunoprecipitation, the cells were treated with Pierce™ Classic Magnetic IP/Co-IP Kit (#88804, Thermo Fisher Scientific, Shanghai, China). According to the manufacturer’s instructions, the cells were carefully removed and washed with cold PBS. Hard immunoprecipitation (IP) lysis was added to the cells and incubated on ice for 5 min with periodic mixing. Next, the lysates were transferred to a microcentrifuge tube and centrifuged at ~13,000 *g* for 10 min to pellet the cell debris. Cell lysates were combined with 5–10 μg of IP antibody per sample in a microcentrifuge tube and incubated overnight at 4°C to form the immune complex. The IP antibodies were as follows: anti-TAFI p48 (TAF1A) (1:200, #sc-393600), anti-RRN3 (1:100, #ab112052), and anti-UBF (1:200, #sc-13125). Then, pre-washed magnetic beads were added and incubated at room temperature for 1 h with mixing. The beads were collected with a magnetic stand and washed three times. Next, 100 μL of Elution Buffer was added to elute the immune complex. Finally, the samples were mixed with Lane Marker Sample Buffer and analyzed by Western blotting with indicated antibodies.

### ChIP and ChIP-PCR

For chromatin immunoprecipitation (ChIP), the cells were treated with SimpleChIP^®^ Enzymatic Chromatin IP Kit (Magnetic Beads) (#9003, Cell Signaling Technology, Danvers, MA, USA). First, the cells were treated with 1% formaldehyde for 10 min for chromatin crosslinking and subsequently with glycine for 5 min for neutralizing, according to the manufacturer’s instructions, and then washed with cold PBS and collected on ice. Acquired chromatin was precipitated with the indicated antibody at 4°C overnight. The antibodies were as follows: anti-POLR1A (1:50, #24799), anti-TAFI p48 (TAF1A) (1:30, #sc-393600), and anti-TBP (10 µg for 25 µg of chromatin, #ab818). Immunoprecipitated products were collected after incubation the next day. The beads were washed, and bound chromatin was eluted and centrifuged. Chromatin was then digested with RNase and proteinase K. After DNA purification, the binding site was evaluated using qPCR with Roche LightCycler^®^ 480 Quantitative PCR System (Indianapolis, IN, USA). The qRT-PCR primers are listed in **Table 2**.

**Table 2:** Sequence of primers used for ChIP-qPCR.

rDNA region	position	forward	position	reverse
upstream	-988	GCTTCTCGACTCACGGTTTC	-798	GGAGCTCTGCCTAGCTCACA
upstream	-410	GATCCTTTCTGGCGAGTCC	-272	GGAGCCGGAAGCATTTTC
promoter	-48	GAGGTATATCTTTCGCTCCGAGTC	-14	CAGCAATAACCCGGCGG
5’ETS	851	GAACGGTGGTGTGTCGTT	961	GCGTCTCGTCTCGTCTCACT
18S	4013	AAACGGCTACCACATCCAAG	4148	CCTCCAATGGATCCTCGTTA
28S	10319	GAACTTTGAAGGCCGAAGTG	10450	ATCTGAACCCGACTCCCTTT
IGS	18499	TGGTGGGATTGGTCTCTCTC	18572	CAGCCTGCGTACTGTGAAAA

### Immunofluorescence and FISH

The cells were fixed in a 4% paraformaldehyde suspension for immunofluorescence and spun onto slides. Then, they were permeabilized with 0.5% PBS-Triton X-100 for 10 min and blocked with 5% goat serum for 1 h. Next, the slides were incubated overnight with indicated antibodies at 4°C followed by the appropriate secondary antibody, either 488 conjugated goat anti-mouse IgG (ab150120, Abcam, Cambridge, UK) or 594 conjugated goat anti-mouse IgG (ab150077, Abcam, Cambridge, UK). The primary antibodies were as follows: anti-fibrillarin (1:100, #ab5821) and anti-nucleophosmin (1:200, #ab10530). For fluorescence *in situ* hybridization (FISH), the cells were treated with the RNA FISH Probe Kit (GenePharma, Shanghai, China). First, they were fixed in a 4% paraformaldehyde suspension and spun onto slides, according to the manufacturer’s instructions, and 100 μL 0.1% Buffer A was added and incubated for 15 min at room temperature. Buffer A was removed and washed twice with PBS; 100 μL of 2× Buffer C was added and incubated for 30 min at 37°C. The probe mixture was prepared, and Buffer C was discarded. Then, 100 μL of the denatured probe mixture was added and set overnight at 37°C for hybridization in a dark place. Next, the probe mixture was removed, and 100 μL of 0.1% Buffer F was added and washed for 5 min. Subsequently, 2× and 1× Buffer C were added. The slides were mounted in Antifade Reagent with DAPI (#8961, Cell Signaling Technology, Danvers, MA, USA) and underwent microscopy. Quantitation was performed using ImageJ software (National Institutes of Health, Bethesda, MD, USA).

### Real-time PCR

The total RNA was extracted using TRIzol reagent (#DP424, Tiangen Biotech, Beijing, China), according to the manufacturer’s instructions. Purified RNA was reverse transcribed to cDNA using PrimeScript™ RT Master Mix (#RR036A, Takara Biomedical Technology, Beijing, China). qRT-PCR was then performed using SGExcelFast SYBR Mixture (#B532955-0005, Sangon Biotech, Shanghai, China) following standard reaction conditions on Roche LightCycler^®^ 480 Quantitative PCR System (Indianapolis, IN, USA). The relative expression of target RNA was calculated using the 2^−ΔΔCt^ method and normalized by the housekeeping gene β-actin level. The qRT-PCR primers are listed in **Table 3**.

**Table 3:** Sequence of primers used for qRT-PCR.

Name	Forward	Reverse
pre-rRNA	GCTGACACGCTGTCCTCTG	TCGGACGCGCGAGAGAAC
β-actin	AGAGCTACGAGCTGCCTGA	AGCACTGTGTTGGCGTACAG

### Tumor xenograft

SMMC-7721 cells (3 × 10^6^ cells/mouse) or SMMC-7721 cells transduced with lentivirus containing shRNA targeting TAF1B were mixed with 100 μL of serum-free culture medium and subcutaneously injected into the nude mice. Tumor size was measured every 5 days using a digital caliper. Tumor volume was calculated using the following formula: tumor volume = (Width^2^ × Length)/2. The mice were euthanized until the most considerable tumor volume was over 800 mm^3^. The tumor burdens were weighed and processed for further evaluation.

### Bioinformatic analysis

The TAF1B in human patient samples was analyzed using the web-based tool Gene Expression Profiling Interactive Analysis (GEPIA; http://gepia.cancer-pku.cn) using The Cancer Genome Atlas (TCGA) data. Overall survival (OS) and disease-free survival (DFS) were plotted using the Kaplan–Meier survival analysis. The group cutoff was set as Cutoff-High (%) = 50% and Cutoff-Low (%) = 50%, the confidence interval was 95%, and data were considered statistically different if log-rank p-value <0.05. The correlation was calculated using Pearson’s correlation coefficient. p < 0.05 was considered significant.

### Statistical analysis

Statistical analyses were performed using IBM SPSS Statistics 26 software (Armonk, NY, USA) and GraphPad Prism 9.1. Values were calculated using Student’s two-tailed t-test or two-way ANOVA. The p-values were expressed as follows: *p < 0.05; **p < 0.01; n.s., no significance.

## Results

### TAF1B is overexpressed in HCC and is associated with worse clinical outcome

**Figure 1:**
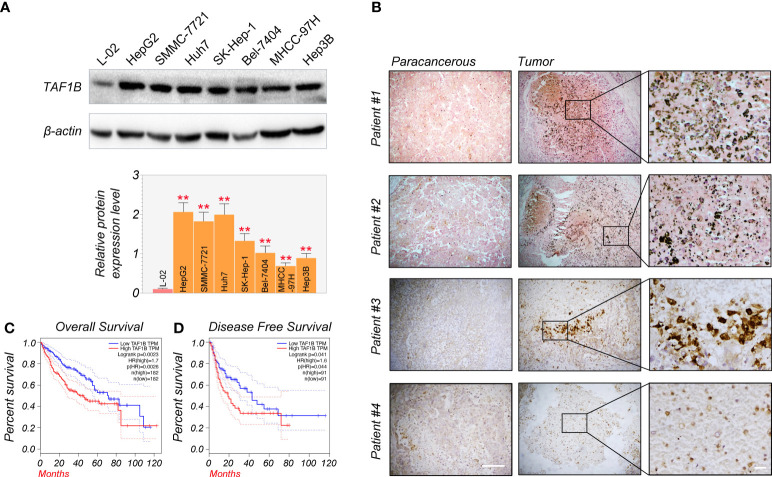
TAF1B overexpression is substantially related to poor survival and clinical outcomes in HCC. **(A)** TAF1B was highly expressed in HCC cell lines than in L-02 cell lines by WB assays (n = 3). **(B)** TAF1B expression in the cancerous tissue and paracancerous tissues from HCC patients. Scale bar, 200 µm. **(C, D)** The analysis of overall survival and disease-free survival of HCC patients stratified by TAF1B expression in TCGA. Data are shown as means ± SD (n = 3). **p < 0.01. HCC, hepatocellular carcinoma; WB, Western blotting; TCGA, The Cancer Genome Atlas.

First, Western blotting detected TAF1B expression in normal human liver cells (L-02) and HCC cell lines (HepG2, SMMC-7721, Huh7, SK-Hep-1, Bel-7404, MHCC-97H, and Hep3B). We found that the expression of TAF1B protein was significantly increased in HCC cell lines compared to L-02 cell lines (**Figure 1A**). We examined the expression of TAF1B in four HCC patients by immunohistochemistry staining. The results indicated a significant increase in TAF1B staining intensity in tumor tissues compared to paracancerous tissues (**Figure 1B**). Furthermore, we analyzed the correlation between TAF1B expression and the clinical outcome of HCC patients. We found that high expression of TAF1B not only shortened the overall survival but also reduced the disease-free survival in HCC patients (**Figures 1C, D**).

### Depletion of TAF1B induces apoptotic cell death in HCC

**Figure 2:**
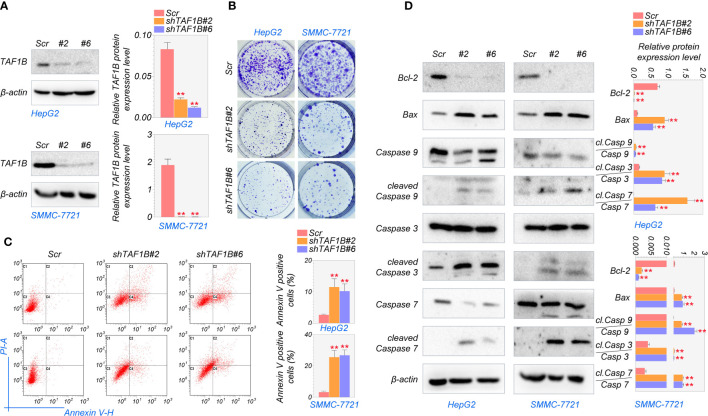
Knockdown of TAF1B induces apoptotic cell death in HCC. HepG2 and SMMC-7721 cells were transduced with lentiviruses expressing either control (scramble) or shRNAs (#2 and #6) targeting TAF1B. **(A)** The efficiency of shRNA mediated knockdown of TAF1B in HepG2 and SMMC-7721 cells. **(B)** Clone formation assay detected cell proliferation after the knockdown of TAF1B in HepG2 and SMMC-7721 cells. The representative images were taken 3 days after transduction. Scale bar, 100 µm. **(C)** The apoptosis of HCC cells was analyzed by flow cytometry. Right, the population of apoptotic cells was calculated as Annexin V-positive cells. **(D)** Western blotting analysis of signaling cascade (BCL-2, BAX, CASP9, cleaved-CASP9, CASP3, cleaved-CASP3, CASP7, and cleaved-CASP7) involved in apoptotic cell death. Cell lysates were collected 3 days after transduction. The band intensity was calculated by ImageJ software and marked. Scr, scramble; #2, sh*TAF1B*#2; #6, sh*TAF1B*#6. Data are shown as means ± SD (n = 3). **p < 0.01. HCC, hepatocellular carcinoma.

Next, we investigated whether TAF1B is crucial for the survival of hepatocellular cancer cells. To inhibit the expression of *TAF1B*, we tested several shRNA targeting *TAF1B* and finally chose #2 and #6 with the best knockdown efficiency for subsequent experiments (**Figures 2A**, **S1**). As shown in **Figure 2B**, the colony formation experiments noted that knockdown *TAF1B* significantly reduced the cell population in HepG2 and SMMC-7721 cells. To further characterize the effect of TAF1B on HCC cells, we examined cell apoptosis. Flow cytometry analysis showed that depletion of TAF1B induced prominent cell apoptosis revealed by counting the Annexin V-positive cells (**Figure 2C**). Then, we examined the marker proteins involved in cell apoptosis, and WB experiments revealed that TAF1B depletion significantly downregulated BCL-2 expression and upregulated BAX, cleaved-CASP9/CASP9, cleaved-CASP3/CASP3, and cleaved-CASP7/CASP7 in HepG2 and SMMC-7721 cells (**Figure 2D**). These discoveries suggested that TAF1B depletion effectively inhibited the viability of hepatocellular carcinoma cells and significantly promoted apoptosis.

### TAF1B deficiency represses the transcriptional function of RNA polymerase I

**Figure 3:**
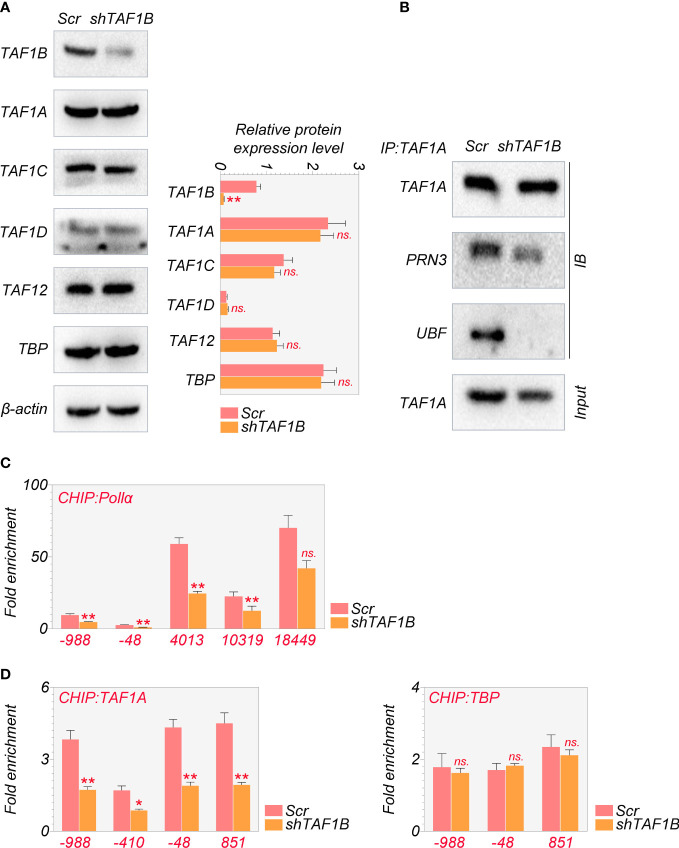
Depletion of TAF1B impairs the transcriptional function of RNA polymerase I HepG2 cells were transduced with lentiviruses expressing scramble or TAF1B shRNA#6. The cell lysates were collected 3 days after transduction. **(A)** The abundance of the components of the SL-1 complex was examined by Western blotting. **(B)** TAF1A-specific antibody was used to immunoprecipitate endogenous TAF1A from HepG2 cell lysates with indicated treatment. The binding of RRN3 and UBF with TAF1A was examined by Western blotting. **(C, D)** The binding of TAF1A, TBP, and RNA polymerase Iα to the rDNA promoter was analyzed by ChIP-qPCR. The positions of amplification primers were indicated numerically. Data are shown as means ± SD (n = 3). *p < 0.05; **p < 0.01; n.s,, no significance; ChIP, chromatin immunoprecipitation.

As mentioned above, the PIC, which consists of SL-1, RRN3, and UBF, was reported to be crucial for cell survival (18). Given that TAF1B is a component of SL-1, we hypothesized that TAF1B depletion caused cell death by influencing the PIC’s function. We examined the abundance and the interaction of the major components of the PIC. The results showed that the protein levels of TAF1A, TAF1C, TAF1D, TAF12, and TBP were not changed after TAF1B knockdown by WB assays (**Figure 3A**). However, the results of co-immunoprecipitation (Co-IP) experiments demonstrated that TAF1A would no longer bind to UBF. In contrast, the binding between TAF1A and RRN3 did not change significantly when TAF1B was removed (**Figure 3B**). Upon TAF1B depletion, not only the interaction among these protein components of PIC was impaired, but the binding of PIC TAF1A with the rDNA promoter was also diminished as revealed by ChIP assays (**Figure 3C**). Moreover, we found that the binding of Pol Iα (POLR1A), the large unit of Pol I, with rDNA was prominently attenuated after TAF1B inhibition. Nevertheless, the binding of TBP to rDNA was not significantly changed (**Figure 3D**). These observations suggested that the lack of TAF1B impaired the transcription function of Pol I in HCC cells.

### TAF1B repression causes nucleolus stress and decreases the expression of pre-rRNA

Reduced ribosome synthesis due to weakened Pol I transcriptional action ultimately causes nucleolar stress. The disturbance of nucleolar structure and changes in nucleolar protein distribution and dynamics are biological hallmarks of rRNA transcription block (6).

**Figure 4:**
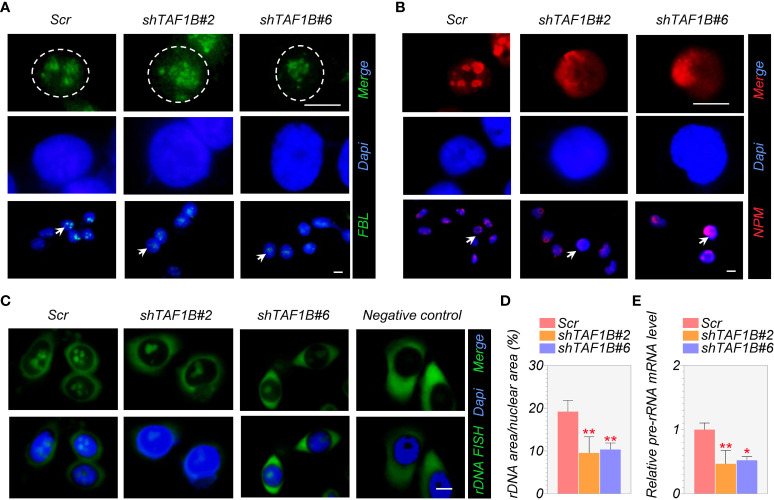
Suppression of TAF1B induces a nucleolar stress response. HepG2 cells were transduced with lentiviruses expressing scramble or TAF1B shRNAs. The cells were fixed and stained by indicated antibodies 3 days after transduction. **(A, B)** Knockdown of TAF1B induced redistribution of fibrillarin (FBL) and nucleophosmin (NPM). Scale bar, 10 µm. **(C)** The representative images of the fluorescence *in situ* hybridization (FISH) for rDNA with DAPI counterstain. Scale bar, 10 µm. **(D)** Quantitation of FISH **(C)**; rDNA area expressed as a percentage of nuclear (DAPI) area (n = 8). **(E)** The expression of pre-rRNA mRNA was determined by qRT-PCR (n = 3). Data are shown as means ± SD. *p < 0.05; **p < 0.01.

Fibrillarin (FBL) and nucleophosmin (NPM) are the primary nucleolar proteins in the stage of proliferating eukaryotic cells and play a significant role in nucleolar stress. When nucleolar stress occurs, the expression levels of these FBL and NPM increase with a transfer of proteins from the nucleus to the cytoplasm. Consistent with this, we discovered in immunofluorescence assays that TAF1B knockdown caused nucleolar structure segregation and changed nucleolar protein localization, including nucleolus fusion and a decrease of FBL staining (**Figure 4A**), and the translocation of granular component proteins NPM to nucleoplasm (**Figure 4B**). In addition, FISH tests revealed a decrease in rDNA transcription when TAF1B was depleted in HepG2 cells compared to the negative control (**Figures 4C, D**). Also, the expression of pre-rRNA mRNA was reduced in TAF1B-depleted HepG2 cells due to the decreased Pol I transcription rate (**Figure 4E**). These data suggested that TAF1B knockdown resulted in nucleolar stress in HepG2 cells.

### Depletion of TAF1B activates the nucleolar surveillance pathway and induces p53-dependent apoptotic cell death of HCC

**Figure 5:**
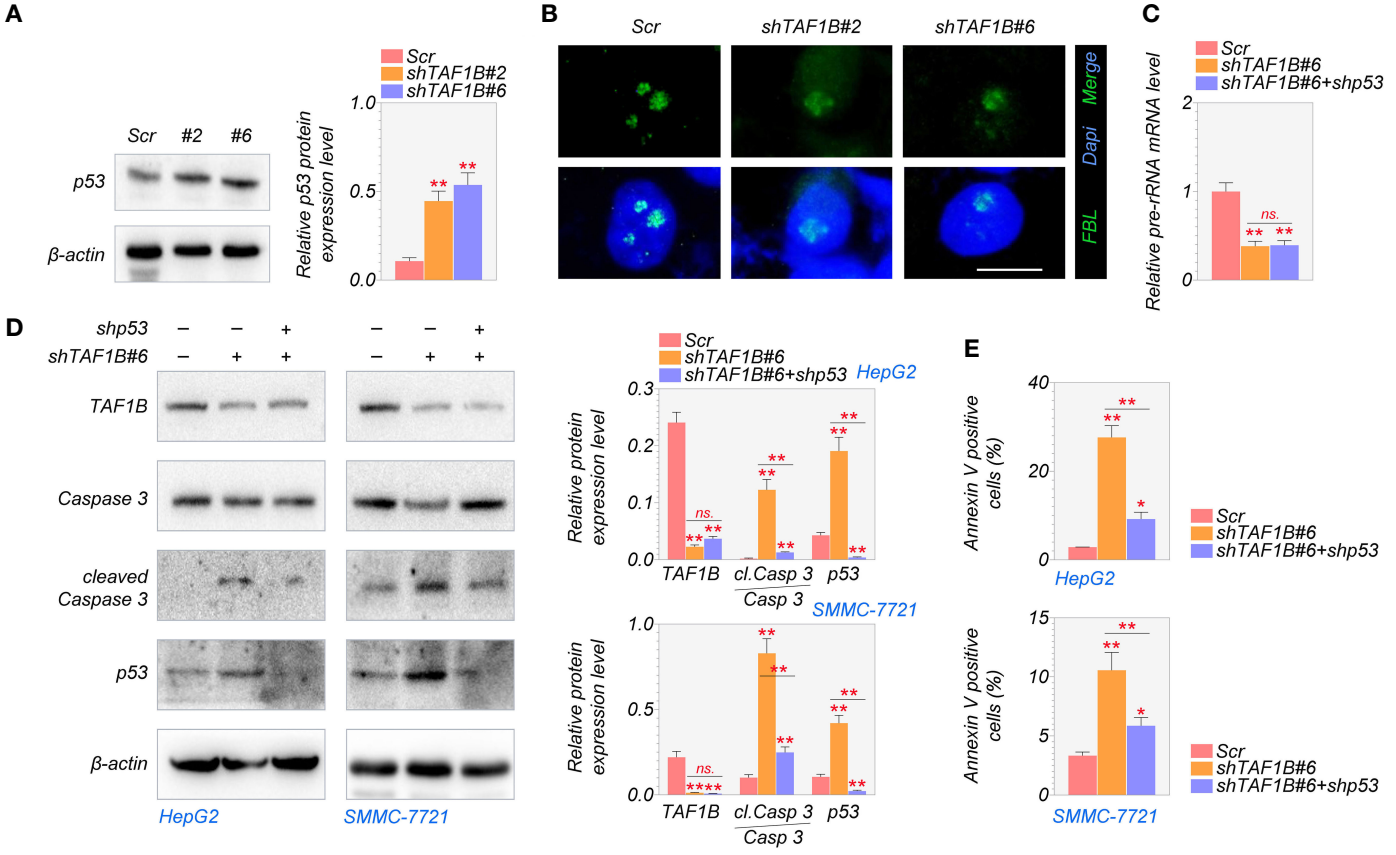
Lack of TAF1B induces p53-dependent apoptotic cell death in HCC. **(A)** The expression of the p53 protein was examined by Western blotting after TAF1B knockdown in HepG2 cells. The cell lysates were collected 3 days after the transduction of TAF1B shRNA. **(B)** Nucleolar stress was shown by FBL distribution. HepG2 cells with indicated manipulation were fixed and stained 3 days after transduction with TAF1B and p53 shRNAs. Representative images are shown. Scale bar, 10 µm. **(C)** The production of pre-rRNA was determined by qRT-PCR (n = 3). **(D, E)** Cell apoptosis was examined by WB assays and flow cytometry after Annexin V/PI co-staining. Data are shown as means ± SD (n = 3). *p < 0.05; **p < 0.01; n.s., no significance. HCC, hepatocellular carcinoma; FBL, fibrillarin; WB, Western blotting.

Numerous studies have shown a strong relationship between p53 activation and nucleolar stress (6). After nucleolar stress, ribosomal proteins are released from the nucleolus and intracellular accumulation of p53 (19). We found that the knockdown of TAF1B increased the expression of p53 protein in HCC cells (**Figure 5A**). To explore whether the nucleolar stress and its associated cell apoptosis are p53-dependent, we inhibited p53 in TAF1B-depleted HepG2 cells. As shown in **Figures 5B, C**, suppression of p53 did not substantially reduce the nucleolar stress induced by TAF1B depletion, indicated by the nucleolar distribution of FBL protein and pre-rRNA expression. These findings showed that in the case of TAF1B knockdown, p53 activation is a subsequent effect of nucleolar stress. Hence, inhibiting p53 does not appreciably reduce nucleolar stress. The examination of cell apoptosis provided further evidence. Suppression of p53 reduced cleavage of caspase-3 in TAF1B-deficient HepG2 and SMMC-7721 cells (**Figure 5D**). Moreover, the proportion of Annexin V-positive cells examined by flow cytometric analysis was decreased after p53 inhibition in TAF1B-deficient HepG2 and SMMC-7721 cells (**Figure 5E**), suggesting repression of p53 protected cell from apoptosis caused by TAF1B deprivation.

### MiR-101 is involved in TAF1B deprivation-induced p53 increase and cell apoptosis

**Figure 6:**
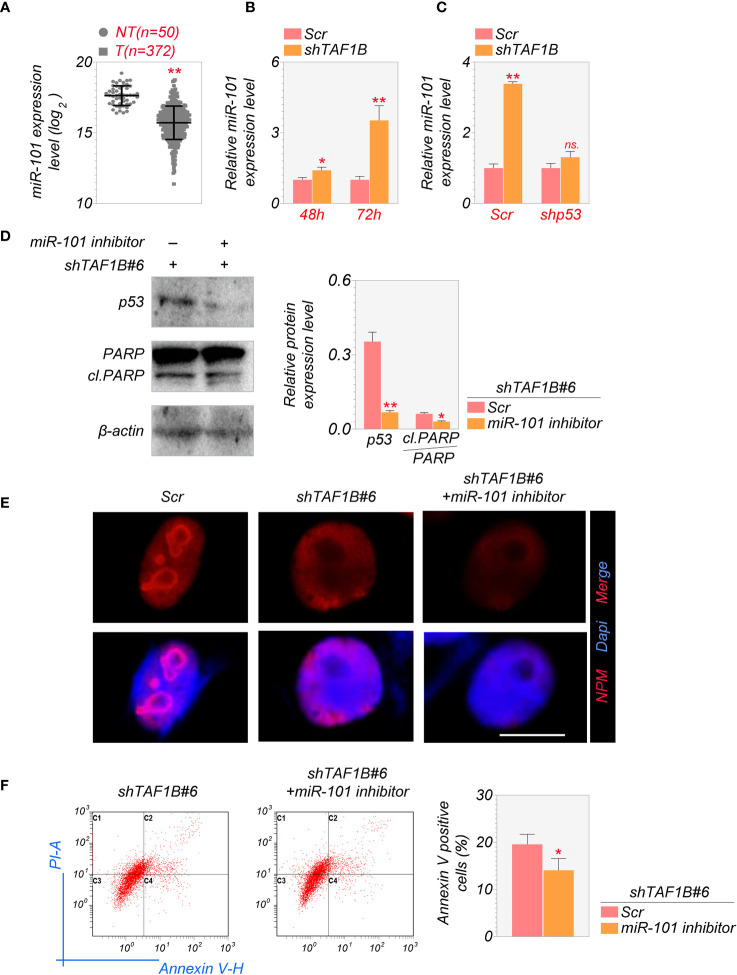
MiR-101 is involved in the induction of nucleolar stress after TAF1B knockdown. **(A)** Expression levels of miR-101 in HCC and non-cancerous liver tissues were determined in the NCI cohort. The miRNA-Seq data were downloaded from The Cancer Genome Atlas (TCGA) Genomic Data Commons (GDC) portal (https://portal.gdc.cancer.gov/repository) and analyzed using RStudio. NT, non-tumor; T, tumor. **(B)** The expression levels of miR-101 in cultured HepG2 cells 48 or 72 h after transduction of sh*TAF1B*-expressing lentiviruses (n = 3). **(C)** qRT-PCR examined the expression of miR-101. As denoted, HepG2 cells were transduced with scramble or shTAF1B#6 plus shp53. RNA was collected 3 days after transduction. **(D)** Inhibition of miR-101 alleviated the increase of the protein levels of p53 and cleaved PARP after TAF1B knockdown. **(E)** The immunofluorescence staining of nucleophosmin (NPM) of HepG2 after miR-101 inhibitor treatment. Scale bar, 10 µm. **(F)** Flow cytometry analyzed the apoptotic cell death of HepG2 cells transduced with shTAF1B#6 after treatment with miR-101 inhibitor. The percentages of Annexin V-positive cells were calculated (n = 3). Scr, scramble; #6, sh*TAF1B*#6. Data are shown as means ± SD. *p < 0.05; **p < 0.01; n.s., no significance; HCC, hepatocellular carcinoma.

It is reported that the activation of miR-101 expression in the p53-miR-101 circuit by inhibiting RNA polymerase I regulates the late stage of nucleolar stress (20). We found that the overall expression level of miR-101 was lower in hepatocellular carcinoma samples than in non-tumor liver tissues through the analysis of TCGA database (**Figure 6A**). Knockdown of TAF1B increased the expression of miR-101 in cultured HepG2 cells (**Figure 6B**). Furthermore, the elevation of miR-101 was p53-dependent, as inhibition of p53 by shRNA abolished TAF1B depletion-induced miR-101 increase by qRT-PCR assays (**Figure 6C**). Accumulation of p53 and enhanced PARP cleavage were detected in cells expressing miR-101 during nucleolar stress, synergistically regulating ribosome biogenesis. Our findings further affirmed that suppression of miR-101 reduced the abundance of p53 and cleaved-PARP proteins (**Figure 6D**). Similar to the observation of p53 inhibition in TAF1B-deficient cells, the treatment of miR-101 did not attenuate nucleolar stress revealed by nucleoplasmic translocation of NPM (**Figure 6E**) but reduced cell apoptosis indicated by Annexin V-PI staining (**Figure 6F**).

### Knockdown of TAF1B promotes hepatocellular apoptotic cell death *in vivo*

**Figure 7:**
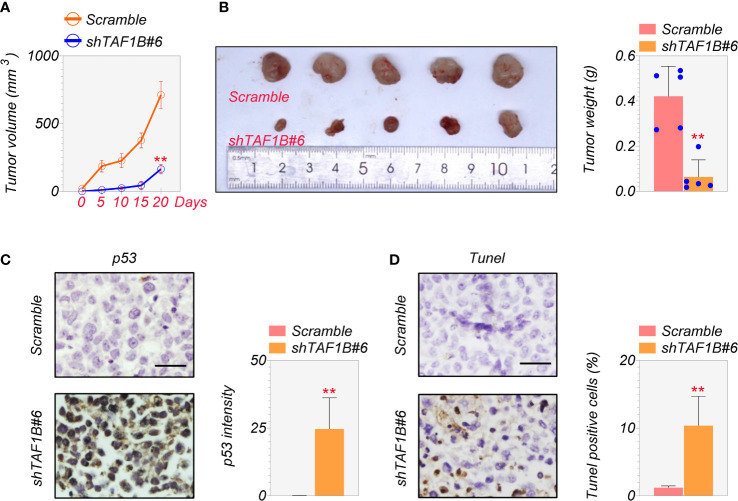
Depletion of TAF1B inhibits HCC tumor growth *in vivo.* The nude mice were randomly divided into two groups. SMMC-7721 cells (3 × 10^6^ cells/mouse) were implanted into the armpits of the mice. **(A)** Tumor growth of SMMC-7721 cells expressing scramble or TAF1B shRNA (n = 5). Tumor size was measured every 5 days. **(B)** Representative image of tumor-burden mice. The image of tumors dissected from tumor-burden mice (left). The measurement of weight of dissected tumors (right) (n = 5). **(C)** The representative images of p53 immunochemical staining in the tumors derived from SMMC-7721 cells (left). Scale bar, 25 µm. Right, quantitation of staining intensity of p53 IHC staining (n = 5). **(D)** TUNEL staining of SMMC-7721-derived tumor. Left, representative images; right, quantitation of TUNEL-positive cells (n = 5). Data are shown as means ± SD. **p < 0.01. HCC, hepatocellular carcinoma; IHC, immunohistochemistry.

Finally, we examined the effect of TAF1B inhibition on tumor development *in vivo*. We inoculated SMMC-7721 cells expressing scramble or TAF1B shRNA into nude mice. As shown in **Figure 7A**, the knockdown of TAF1B significantly reduced the growth of SMMC-7721 cell-derived tumors. In addition, the size and weight of the tumors formed by the tumor cells lacking TAF1B were considerably decreased (**Figure 7B**). Immunostaining and TUNEL assay showed that the expression of p53 and apoptotic cell death significantly increased in TAF1B-depleted tumors (**Figures 7C, D**).

## Discussion

Ribosome biogenesis is a well-known feature of cell development and proliferation. It has lately emerged as an effective cancer therapeutic target. The first critical step in ribosome biogenesis is rRNA transcription by Pol I. As a result, medicines that preferentially target Pol I transcription are emerging as a novel class of anticancer therapeutics. Some selective Pol I inhibitors have shown potential therapeutic benefits. CX-3543, for example, has broad anti-proliferative and apoptotic effects on cancer cells and displayed remarkable anti-tumor growth capabilities in breast and pancreatic cancer xenograft models (21). Surprisingly, the following generation of CX-3543, that is, CX-5461, demonstrated efficacy in human cancer cells with overloaded ribosomal biogenesis compared to normal cells (22, 23). According to our findings of the significant high expression of TAF1B in hepatocellular carcinoma tissues and its poor prognosis, we conclude that targeting TAF1B may give another method of treating malignancies by interfering with the transcriptional activity of Pol I.

TAF1B is a TFIIB-like component of the basal transcription machinery for RNA polymerase I (24). It has been reported to be positioned in the RNAP1-PIC, similar to TFIIB in the RNAP2-PIC (14). Disruption of TAF1B by RNA interference reduced rRNA synthesis, leading to decreased ovarian germline stem cell proliferation in *Drosophila* (25). However, investigations of TAF1B in human diseases are scarce. Similar to a previous study, we observed reduced rRNA production in HCC cells after TAF1B inhibition, suggesting its conserved function in evolution. Clone formation and apoptosis assays revealed that inhibition of TAF1B expression in HCC significantly repressed cell proliferation and promoted cell apoptosis. Considering the crucial function of TAF1B in Pol I transcription, we checked the main transcriptional cofactors of PIC. We discovered that TAF1B depletion mainly affected the binding interaction between TAF1A and UBF to regulate rDNA activity, thus promoting HCC death. Immunofluorescence experiments further confirmed our conjecture that FBL and NPM were transferred from the nucleus to the cytoplasm in TAF1B-depleted HCC, symbolizing nucleolar stress activation.

The p53 tumor suppressor protein is an integration point in response to various cellular stresses. Activating p53 can promote transcription of p21, leading to G1/S growth arrest or BAX-inducing apoptotic cell death (26). Nucleolar stress can provoke cell cycle arrest or apoptosis via p53-dependent and p53-independent signaling pathways. During nucleolar oxidation, NPM undergoes *S*-glutathionylation on cysteine 275. It triggers the dissociation of NPM from nucleolar nucleic acids and promotes NPM binding to HDM2 (27), which blocks the E3 ligase activity of HDM2 and induces p53 accumulation. c-Myc, E2Fs, and SP1 are the major non-p53 TFs that respond to ribosomal stress (6). Under impaired rRNA biosynthesis, free ribosomal proteins RPL5 and RPL11 can form a complex with c-Myc mRNA and recruit microRNAs to repress c-Myc expression, leading to inhibition of cell proliferation through suppression of c-Myc and its target gene expression (28). In this study, we observed that cell apoptosis induced by TAF1B deprivation was p53-dependent. Inhibition of p53 indeed improved TAF1B-induced cell apoptosis, while nucleolar stress was not attenuated, emphasizing that the activation of p53 was triggered by nucleolar stress. Although most activation of the nucleolar stress response relies on p53 activity, some p53 knockout cells induce DNA damage or replication and ribosomal stress in a checkpoint kinase 1 (Chk1) phosphorylation-dependent manner, effectively reducing the proliferation of cancer cells (29). Thus, cell cycle arrest can occur even in the absence of p53.

Various stresses change the biogenesis, modification, and function of miRNAs. However, the investigation of the role of miRNA in nucleolar stress is rare. Our results demonstrated that nucleolus stress was implicated in miR-101 activation and that nucleolus stress-induced miR-101 biogenesis was mediated at the post-transcriptional level. This is consistent with the study of Naoto Tsuchiya et al. (20) that the p53-dependent modulation of miR-101 biogenesis occurs only in the context of nucleolus stress. Meanwhile, our study found that miR-101 was involved in TAF1B depletion-induced p53 accumulation. Inhibition of miR-101 decreased the accumulation of p53. Interestingly, p53 inhibition abolished the enhanced miR-101 expression under TAF1B knockdown. Similar to p53, inhibition of miR-101 moderately attenuated apoptotic cell death but did not improve nucleolar stress, suggesting that both p53 and miR-101 were downstream effectors of the nucleolar stress response pathway.

Taken together, we found TAF1B was crucial for Pol I function and the cancer progression in HCC. Inhibition of TAF1B caused significant nucleolar stress and apoptotic cell death. The activation of the p53-miR-101 circuit was involved in TAF1B-induced cell apoptosis. Targeting TAF1B may serve as a novel approach for HCC treatment.

## Data availability statement

The datasets presented in this study can be found in online repositories. The names of the repository/repositories and accession number(s) can be found in the article/**Supplementary Material**.

## Ethics statement

The animal study was reviewed and approved by Zhejiang Eyong Pharmaceutical Research and Development Center (license number for animal use: SYXK 2021-0033).

## Author contributions

Participated in research design: FZ and H-FC. Conducted experiments: H-FC, D-DG, X-QJ, QW, QZ, and Q-CZ. Performed data analysis: LY, ML, L-FX, HX, M-XQ, JF, and FZ. Wrote or contributed to the writing of the manuscript: FZ, H-FC, and JF. All authors contributed to the article and approved the submitted version.

## Supplementary material

Supplementary Figure 1 The expression of TAF1B protein was examined by western blotting after several TAF1B knockdowns. The cell lysates were collected 3 days after the transduction of TAF1B shRNAs.
