# Supplementary figures and images for: TAF1B depletion leads to apoptotic cell death by inducing nucleolar stress and activating p53-miR-101 circuit in hepatocellular carcinoma
Source: Front Oncol. 2023 Aug 14;13:1203775. doi: 10.3389/fonc.2023.1203775 (PMC10461479; doi:10.3389/fonc.2023.1203775)

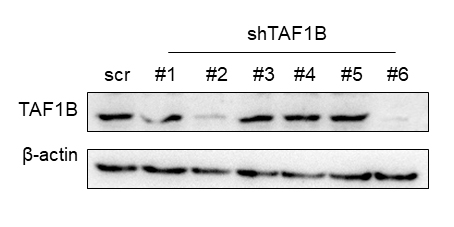

Supplement: Supplementary Figure 1 — The expression of TAF1B protein was examined by western blotting after several TAF1B knockdowns. The cell lysates were collected 3 days after the transduction of TAF1B shRNAs. [file Image_1.jpeg]

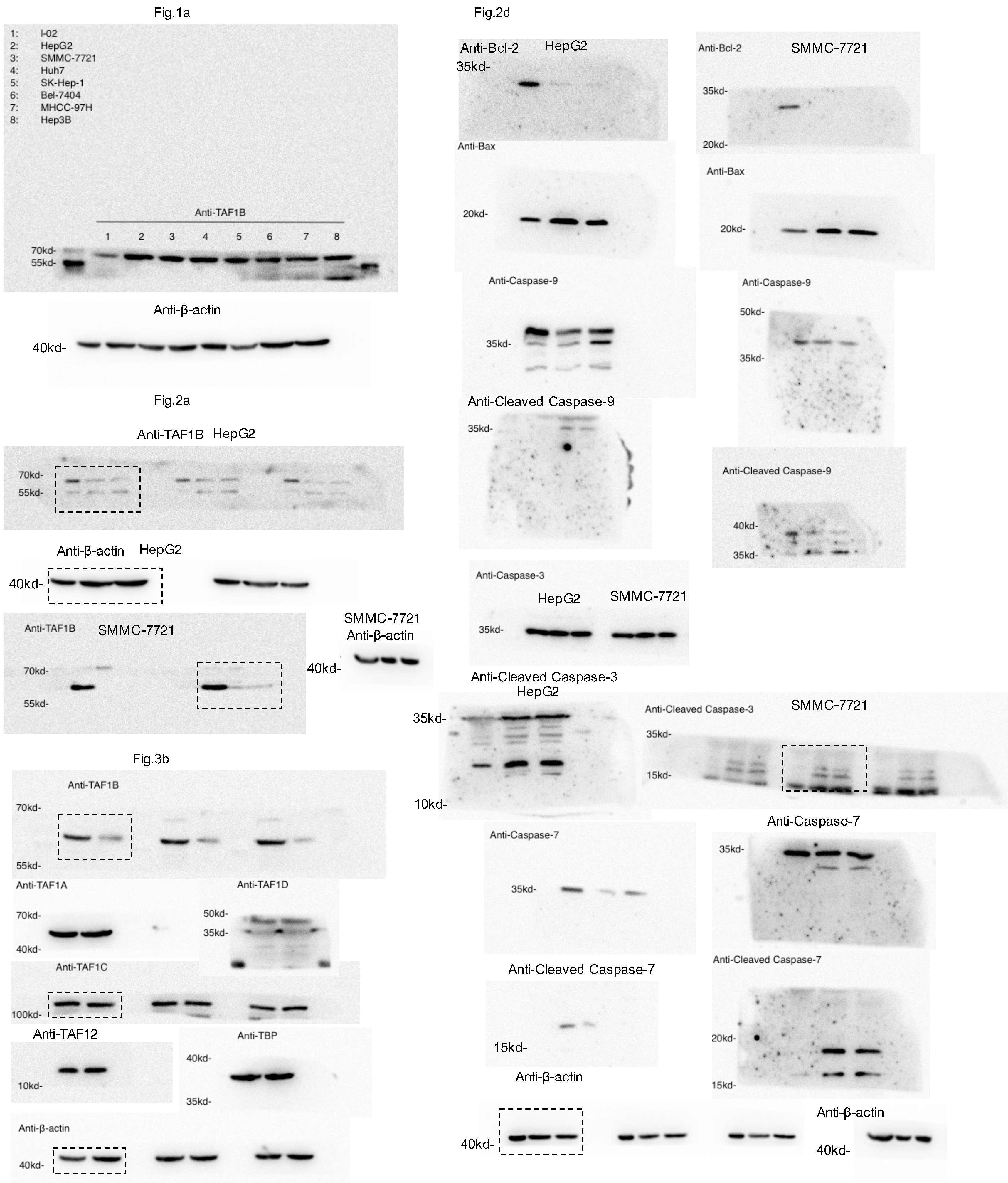

Supplement: Supplementary file 2 [file Image_2.jpeg]

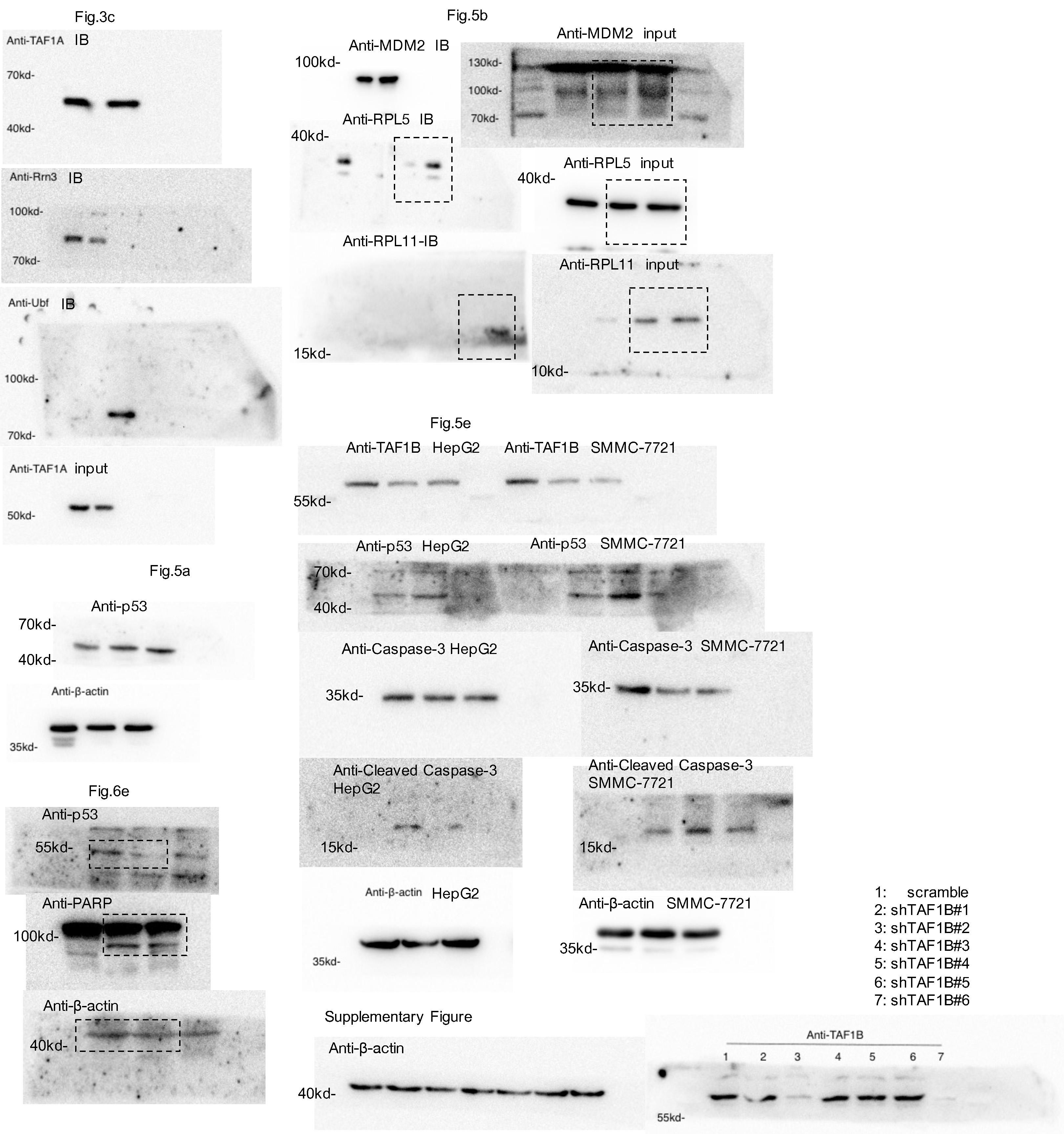

Supplement: Supplementary file 3 [file Image_3.jpeg]

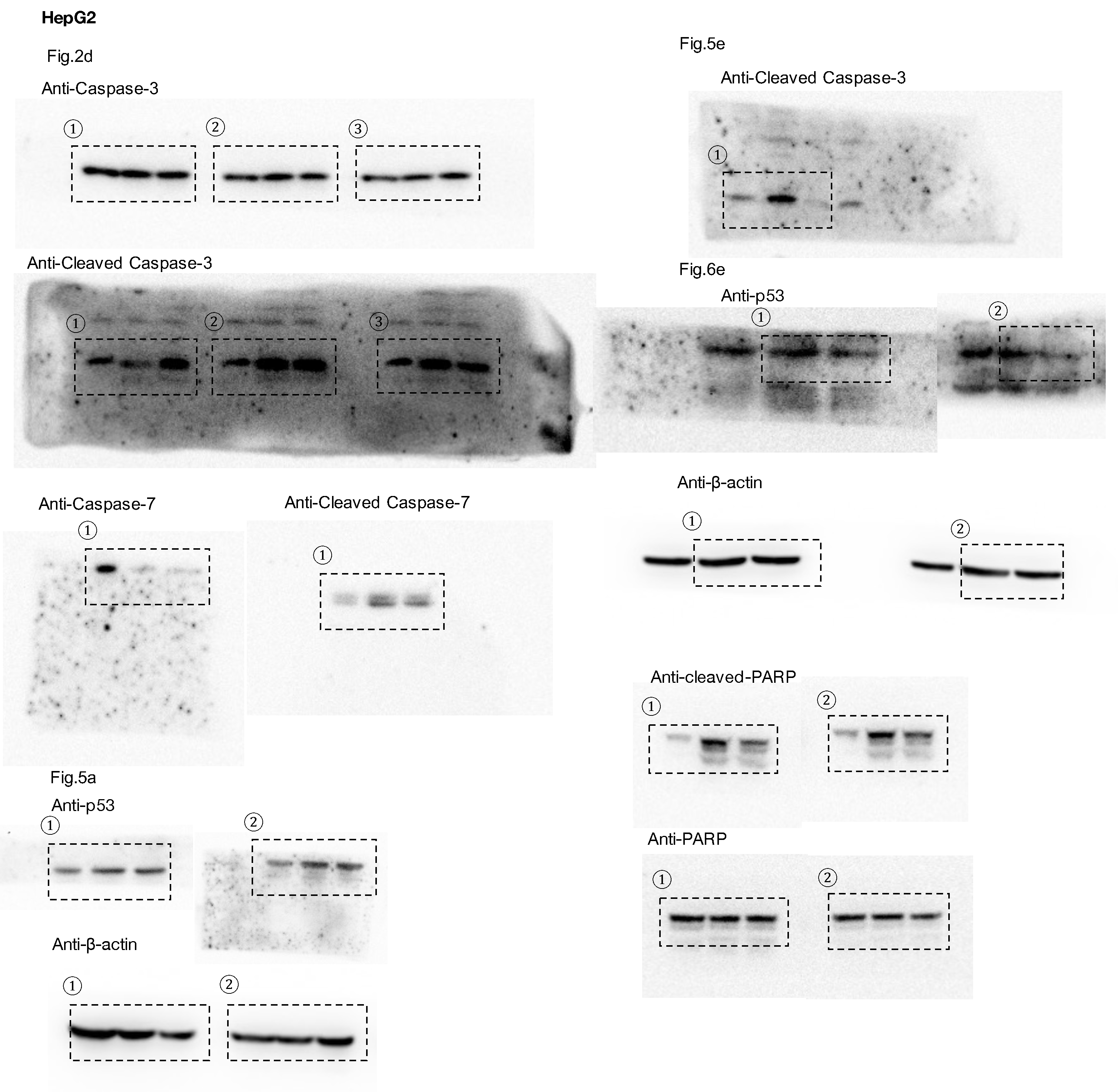

Supplement: Supplementary file 4 [file Image_4.jpeg]
